# Thermal Optimization of a Dual Pressure Goswami Cycle for Low Grade Thermal Sources

**DOI:** 10.3390/e21070711

**Published:** 2019-07-20

**Authors:** Gustavo Guzmán, Lucía De Los Reyes, Eliana Noriega, Hermes Ramírez, Antonio Bula, Armando Fontalvo

**Affiliations:** 1 Department of Mechanical Engineering, Universidad Autónoma del Caribe, Barranquilla 080020, Colombia; 2 AST Ingeniería SAS, Barranquilla 080001, Colombia; 3 Department of Energy, Universidad de la Costa, Barranquilla 080002, Colombia; 4 Department of Mechanical Engineering, Universidad del Norte, Barranquilla 081007, Colombia; 5 Research School of Electrical, Mechanical and Materials Engineering, The Australian National University, Acton, ACT 2600, Australia

**Keywords:** power and cooling, ammonia-water mixture, low-temperature cycle, dual-pressure Goswami cycle

## Abstract

This paper presents a theoretical investigation of a new configuration of the combined power and cooling cycle known as the Goswami cycle. The new configuration consists of two turbines operating at two different working pressures with a low-heat source temperature, below 150 °C. A comprehensive analysis was conducted to determine the effect of key operation parameters such as ammonia mass fraction at the absorber outlet and boiler-rectifier, on the power output, cooling capacity, effective first efficiency, and effective exergy efficiency, while the performance of the dual-pressure configuration was compared with the original single pressure cycle. In addition, a Pareto optimization with a genetic algorithm was conducted to obtain the best power and cooling output combinations to maximize effective first law efficiency. Results showed that the new dual-pressure configuration generated more power than the single pressure cycle, by producing up to 327.8 kW, while the single pressure cycle produced up to 110.8 kW at a 150 °C boiler temperature. However, the results also showed that it reduced the cooling output as there was less mass flow rate in the refrigeration unit. Optimization results showed that optimum effective first law efficiency ranged between 9.1% and 13.7%. The maximum effective first law efficiency at the lowest net power (32 kW) and cooling (0.38 kW) outputs was also shown. On the other hand, it presented 13.6% effective first law efficiency when the net power output was 100 kW and the cooling capacity was 0.38 kW.

## 1. Introduction

Heat recovery is a suitable way to improve energy conversion efficiency in industrial processes and conventional heat-to-power generation plants [1,2]. However, most heat recovery systems are designed for medium- (200–350 °C) and high-temperature (350–500 °C) waste heat sources, and they are not suitable for low-temperature heat sources (100–200 °C). As a consequence, new alternative cycles like the Organic Rankine Cycle (ORC), the Kalina cycle, and the Goswami cycle have been proposed for waste heat recovery and renewable energy use in solar, geothermal, and biomass applications [3,4,5,6,7,8]. These cycles use low-boiling temperature binary mixtures, which exhibit a good thermal match with sensible heat sources and therefore reduce heat transfer irreversibilities [9]. Among these cycles, the Goswami cycle exhibits a higher efficiency as it produces simultaneous power and cooling, by keeping the advantages of the Kalina cycle and the simplicity of the ORC cycle [7].

**Figure 1:**
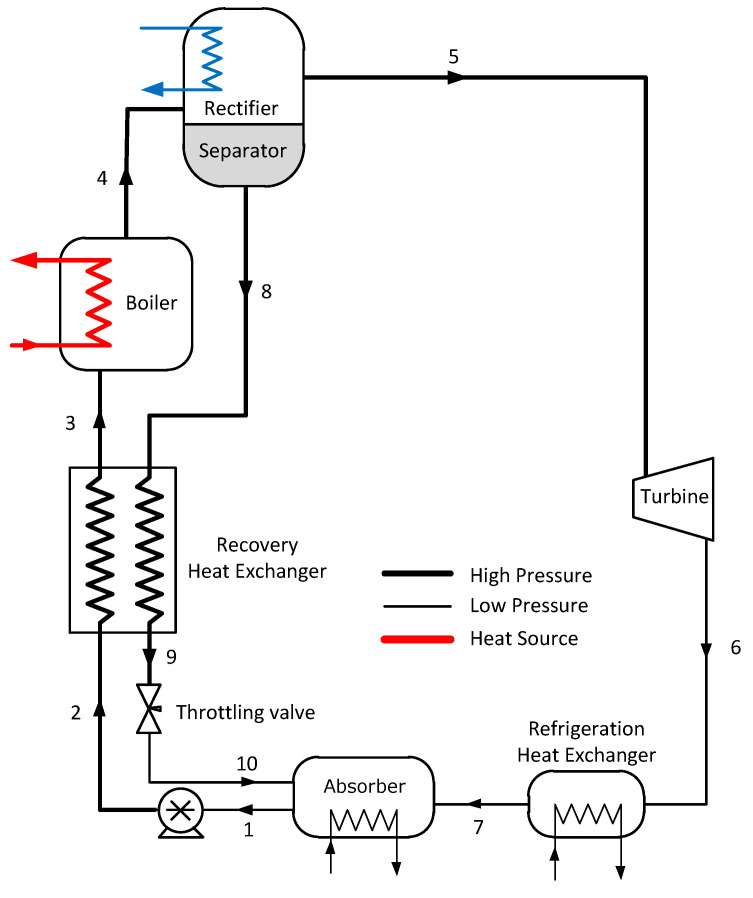
Original single pressure Goswami cycle.

The Goswami cycle (see Figure 1) is a cogeneration cycle that combines a Rankine cycle and an absorption refrigeration cycle to produce either power, cooling, or simultaneous power and cooling. The cycle uses an ammonia-water mixture, which is vaporized and split to obtain a high-concentration ammonia vapor for power production in a Rankine turbine, and a cooling supply in a refrigeration heat exchanger. Since it was proposed, several authors have addressed parametric studies and multivariable optimization to study the effect of design and operational parameters on the performance of the cycle.

Initial publications performed theoretical investigations to identify operating trends by changing key design parameters in the turbine and the heat exchangers. Xu et al. [7] conducted a thermodynamic analysis of a Goswami cycle with internal rectification cooling under idealized conditions. Their study evaluated the effect of the main operation parameters on vapor flow rate at the rectifier, turbine power output, cooling capacity, and thermal efficiency when the absorber ammonia mass fraction was varied between a 0.47- and 0.43-kg NH_3_/kg solution. They concluded that the increase of turbine inlet pressure up to 28 bar hindered vapor production in the rectifier and power output in the turbine, but improved cooling capacity and thermal efficiency. However, if turbine inlet pressure was increased above 28 bar, the performance of the cycle was penalized. They also showed that an increase in boiler temperature boosted thermal efficiency, but penalized cooling capacity.

Hasan et al. [10] carried out a first law and second law analysis of the Goswami cycle when solar heat source temperature was 420 K. They obtained a maximum second law efficiency of 65.8% and showed that the absorber had the largest contribution to cycle irreversibility, but also, the rectifier and the boiler showed a significant contribution. Tamm et al. [11] presented a theoretical and experimental analysis of the Goswami cycle by considering pipeline and pressure drop irreversibilities. Their study showed that pressure losses below 5% in heat exchanger equipment had a negligible effect on the cycle performance. They also presented an experimental study, which verified the expected boiling and absorption processes and supported their theoretical results.

On the other hand, the definition of an appropriate efficiency expression and the use of optimization algorithms have also been studied to maximize simultaneous power and cooling. Vijayaraghavan and Goswami [12] introduced an efficiency expression to weight the cooling production of the cycle and optimized the cycle performance by using a Generalized Reduced Gradient (GRG) method. Their results showed that the weight assigned to refrigeration output had a significant impact on the optimum parameters for the cycle as higher weights reduce optimum cooling capacity, but maximize power output. Martin and Goswami [9] optimized cooling production in the Goswami cycle, by introducing an effective Coefficient Of Performance (COP) that related cooling capacity and the reduction of the potential power production. They showed that the cycle can be optimized for cooling and power, but they did not find an operating point that maximized cooling and power at the same time.

Padilla et al. [13] carried out a parametric study of the Goswami cycle to study the effect of turbine inlet pressure and isentropic efficiency and the absorber ammonia mass fraction on net power output, cooling capacity, and effective first law and exergy efficiencies. They varied the heat source temperature between 90 °C and 170 °C. Their results showed that the maximum effective first law and exergy efficiencies were 20% and 72%, respectively. They also found that the highest efficiency and net power output were obtained when the rectifier and the superheater were removed, but the rectifier was necessary for cooling. Finally, they showed that the superheater improved net power output, but its effect was negligible at high pressure ratios.

Pouraghaie et al. [14] carried out a multi-objective optimization of the thermodynamic performance of the Goswami cycle by using a Pareto approach. The multi-objective algorithm varied turbine inlet pressure, superheater temperature, and condenser temperature, to maximize simultaneously turbine power output, cooling capacity, and first law efficiency. They showed that higher turbine inlet pressures and superheater temperatures lead to higher turbine power outputs, but penalize thermal efficiency and cooling capacity. They also showed that the increase in condensation temperature had a negative impact on cooling capacity and thermal efficiency.

Demirkaya et al. [15] conducted a thermodynamic optimization of the Goswami cycle by using a genetic algorithm. Their study evaluated the effect of lo- and medium-temperature heat sources on turbine power output, cooling capacity, and effective first law and exergy efficiencies. Their study covered boiler temperatures from 70–250 °C. The optimization results concluded that optimum effective first law efficiency can be improved if net power is increased and cooling output is reduced.

Variations of the Goswami cycle have also been proposed to improve the cycle performance. Fontalvo et al. [16] presented an exergy analysis of two configurations proposed by Demirkaya et al. [17]. They showed that internal rectification cooling reduced the total exergy destruction of the cycle, and the use of the superheater reduced the exergy destruction of the cycle at high turbine efficiency values. Demirkaya et al. [18] found promising results when multi-stage turbines were used. The results showed that multi-stage turbines increased thermal performance compared to single-stage turbines when heat source temperatures were above 200 °C and had a negligible effect when heat source temperature was below 175 °C. The results in [18] exhibited no cooling output when boiler temperatures were above 100 °C, and most of the above-mentioned publications were conducted for boiler and rectifier temperatures below 100 °C. Therefore, there is still a gap in the study of the effect of operation parameters on the Goswami cycle performance, especially on the cooling output. In addition, as the cycle’s cooling and power output can be obtained simultaneously, but the increase of the cooling capacity sacrifices net power output, the use of an additional working pressure for power production and a middle pressure loop for power and cooling could increase the Goswami cycle’s performance.

This paper presents a new configuration of the Goswami cycle, which consists of two single-stage turbines operating at two different working pressures from a low-heat source temperature, below 160 °C. The effect of boiler temperatures, rectifier temperatures, and absorber ammonia mass fraction on net power output, cooling capacity, and effective first law and exergy efficiencies is also investigated, by conducting a parametric study and running a multi-objective metaheuristic optimization algorithm. Finally, optimum performance metrics are reported and discussed.

## 2. Materials and Methods

### 2.1. Description of the Cycle

**Figure 2:**
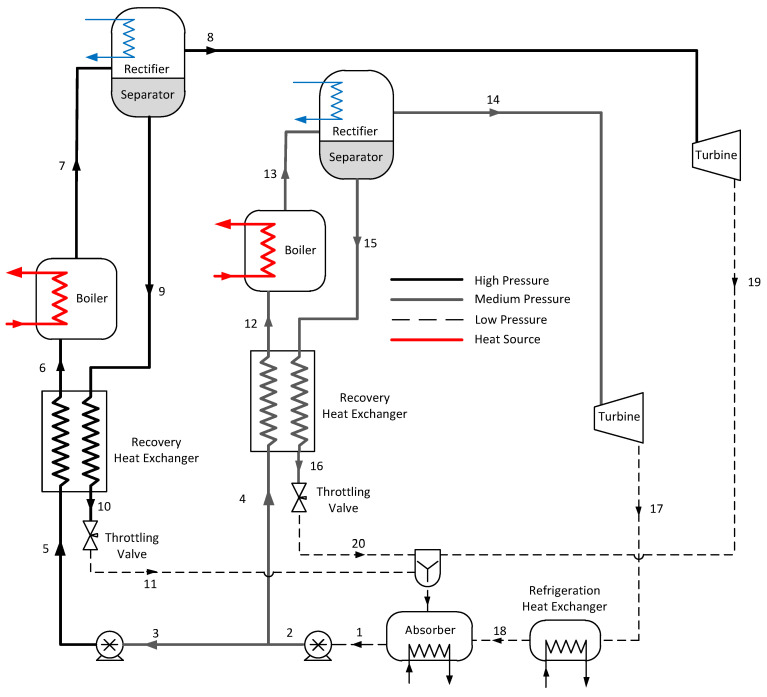
Proposed dual-pressure Goswami cycle.

The proposed cycle modifies the original Goswami cycle by using two operation pressures as shown in Figure 2. A strong ammonia-water mixture leaving the absorber as saturated liquid (Stream 1) was pumped to the system middle pressure (Stream 2) and split into two streams (3 and 4). Then, Stream 3 was pumped to the system at high pressure (Stream 5) and preheated (Stream 6) in a recovery heat exchanger before it was vaporized (Stream 7). This vaporized mixture leaving the boiler was split into a high-pressure ammonia-rich saturated vapor (State 8) and a high-pressure ammonia weakly-saturated liquid (State 9) in the separator-rectifier column. The liquid in Stream 9 was cooled down and throttled while the saturated vapor in Stream 8 was expanded in the high-pressure turbine to the system at low pressure, before both streams were rejoined in the absorber. The ammonia-water in Stream 4 was preheated (Stream 12), vaporized (Stream 13), and split into a mid-pressure ammonia-rich saturated vapor (Stream 14) and a mid-pressure ammonia weakly-saturated liquid (Stream 15). The saturated vapor in Stream 14 was expanded to the system at low pressure (Stream 17) in the mid-pressure turbine, and it was used as a refrigerant fluid in the refrigeration heat exchanger before it was returned to the absorber. Finally, Stream 15 was cooled down in the mid-pressure recovery heat exchanger and throttled, before it returned to the absorber along with Streams 11, 20, and 19.

### 2.2. Thermodynamic Analysis

This paper focuses on evaluating the Goswami cycle performance when it recovers energy from a thermal source between 110 °C and 160 °C, by using solar thermal collectors with hot water as the heat transfer fluid. For this purpose, the first and second law of thermodynamics were applied to each component of the cycle. Energy and exergy balances were conducted to determine the performance of the combined cycle in terms of the following performance metrics: net power output (${\dot{W}}_{\mathrm{net}}$), cooling capacity (${\dot{Q}}_{cool}$), effective first law efficiency ($\eta_{\mathrm{th}}$), and effective exergy efficiency ($\eta_{\mathrm{ex},\mathrm{eff}}$). These metrics were selected to quantify the cycle performance from an energy accounting point of view, but also to determine the quality of the energy conversion process by introducing the concept of entropy generation, which is linked to the exergy efficiency metric.

#### 2.2.1. Energy and Exergy Balances

Each component in the two studied configurations was assumed as a control volume working under steady-state operation. A general mass balance (Equation (1)), an ammonia mass balance (Equation (2)), and an energy balance (Equation (3)) were applied to each component of the cycle [19].

(1) $$\sum_{i}{\dot{m}}_{i}-\sum_{e}{\dot{m}}_{e}=0$$

(2) $$\sum_{i}{\dot{m}}_{i}x_{i}-\sum_{e}{\dot{m}}_{e}x_{e}=0$$

(3) $$\sum_{i}{\dot{m}}_{i}h_{i}-\sum_{e}{\dot{m}}_{e}h_{e}+\sum_{j}{\dot{Q}}_{j}-{\dot{W}}_{cv}=0$$

where $\dot{m}$ is the stream mass flow rate, *x* is the stream ammonia mass fraction, *h* is the stream specific enthalpy, ${\dot{Q}}_{j}$ is the heat transfer input to the control volume, and ${\dot{W}}_{cv}$ is the power output of the control volume.

An exergy balance (Equation (4)) was conducted to determine the exergy destruction of each component of the studied configurations [19].

(4) $$\sum_{j}{\dot{E}}_{qj}-{\dot{W}}_{cv}+\sum_{i}{\dot{m}}_{i}e_{fi}-\sum_{e}{\dot{m}}_{e}e_{fe}-{\dot{E}}_{d}=0$$

with:

$${\dot{E}}_{qj}=\left(1-T_{o}/T_{j}\right){\dot{Q}}_{j}$$

$$e_{f}=h-h_{o}-T_{o}\;\left(s-s_{o}\right)+V^{2}/2+g\;z$$

where ${\dot{E}}_{qj}$ is the exergy input due to heat transfer, $e_{f}$ is the stream specific exergy, and ${\dot{E}}_{d}$ is the exergy destruction.

#### 2.2.2. Performance Metrics

The cascade analogy [12] provides the suitable efficiency terms to measure the performance of the combined cycle based on the useful energy of the cycle. Net power output was calculated by means of Equation (5), while cooling output was determined from Equation (6), and effective first law efficiency was calculated from Equation (7).

(5) $${\dot{W}}_{\mathrm{net}}=\sum {\dot{W}}_{turb}-\sum {\dot{W}}_{pump}$$

(6) $${\dot{Q}}_{cool}={\dot{m}}_{\mathrm{cf}}\left(h_{\mathrm{cf},\;i}-h_{\mathrm{cf},\;o}\right)$$

(7) $$\eta_{\mathrm{th}}=\frac{{\dot{W}}_{\mathrm{net}}+{\dot{E}}_{\mathrm{cool}}/\eta_{\mathrm{II},\mathrm{ref}}}{{\dot{Q}}_{h}}$$

In the above equation, ${\dot{E}}_{\mathrm{cool}}$ is the exergy associated with the cooling output. To account for the heat transfer irreversibilities in the refrigeration heat exchanger, the exergy change of the chilled fluid was considered and determined in Equation (8).

(8) $${\dot{E}}_{\mathrm{cool}}={\dot{m}}_{\mathrm{cf}}\left[h_{\mathrm{cf},\;i}-h_{\mathrm{cf},\;o}-T_{0}\left(s_{\mathrm{cf},\;i}-s_{\mathrm{cf},\;o}\right)\right]$$

Effective exergy efficiency is given as:

(9) $$\eta_{\mathrm{ex},\mathrm{eff}}=\frac{W_{\mathrm{net}}+E_{\mathrm{cool}}/\eta_{\mathrm{II},\mathrm{ref}}}{E_{\mathrm{hs},i}-E_{\mathrm{hs},o}}$$

In the previous equation, the denominator is the change in exergy of the heat source, which is equivalent to the exergy input. The exergy input represents the maximum amount of useful work that can be obtained from the heat source.

### 2.3. Simulation Details

A computer simulation program was written in MATLAB^®^ with the mass and energy balances of the cycle. Furthermore, the thermodynamic properties of the ammonia-water mixture were calculated using the correlations for thermodynamic properties proposed by Xu and Goswami [20]. The validation of these correlations was demonstrated by the authors in a previous publication [16], where it was compared with the experimental data obtained by Tillner-Roth and Friend [21].

#### 2.3.1. Parametric Study

A parametric study was proposed to evaluate the effect of boiler and rectifier temperatures and absorber ammonia mass fraction ($x_{1}$) on the performance of the single-pressure Goswami cycle in Figure 1. These variables define the cycle pressure limits and the vapor production in the rectifier column, hence they define the turbine power output and the cycle cooling capacity [13]. These parameters were varied according to Table 1. For each boiler temperature, rectifier temperature, and absorber ammonia-mass fraction combination in Table 1, the cycle high pressure was varied between the boiler dew and bubble pressures to optimize each performance metric separately.

The remaining simulation parameters presented in Table 2 were selected based on previous simulations presented by Demirkaya et al. [18] to obtain a similar basis to analyze the results. In addition, the following assumptions were employed in the parametric study:

- The system low pressure was calculated as a function of the ammonia mass fraction at the absorber outlet, $x_{1}$, and the absorber temperature to obtain a saturated liquid leaving the absorber.
- The boiling conditions were completely specified, i.e., boiling temperature, pressure, and solution concentration were provided as inputs.
- The effectiveness value was used for the heat recovery heat exchanger, while the pinch point limitation was 10 °C for the boiler and refrigeration heat exchangers.
- Superheating was not considered in this simulation, since superheating reduces cooling output.
- Pressure drops were neglected, as Tamm et al. [11] showed that pressure losses below 5% had a negligible effect on the cycle performance. Therefore, this study suggested that all heat exchanger and piping systems must be designed to achieve pressure losses below 5%.

For the dual-pressure configuration in Figure 2, boiler and rectifier temperatures, as well as absorber ammonia mass fraction ($x_{1}$), and the high-pressure loop to mid-pressure loop mass flow rate ratio (${\dot{m}}_{3}/{\dot{m}}_{2}$) were varied according to Table 3. As in the single-pressure configuration, these variables were chosen because they define the vapor production in the rectifier columns. Therefore, they define power production in each turbine and the cycle cooling capacity. The remaining design parameters are defined in Table 2. For each parameter combination in Table 3, the cycle’s high and middle pressure were varied to optimize each performance metric separately.

#### 2.3.2. Optimization Study

The performance metrics ${\dot{W}}_{net}$, ${\dot{Q}}_{cool}$, and $\eta_{I,\mathrm{eff}}$ were not continuous in the domain of the rectifier and boiler temperatures of the dual-pressure configuration. The operation conditions that maximize net power output were not the same that maximize the cooling capacity and effective first law efficiency. This behavior was influenced by rectifier temperature: At a given boiler pressure, the ammonia mass fraction at the rectifier outlet was increased when rectifier temperature decreased, which reduced the turbine outlet temperature and allowed the cooling effect at the refrigeration unit. However, this hindered the rich vapor production in the rectifier and reduced the mass flow rate across the turbine, which reduced the turbine power output.

**Figure 3:**
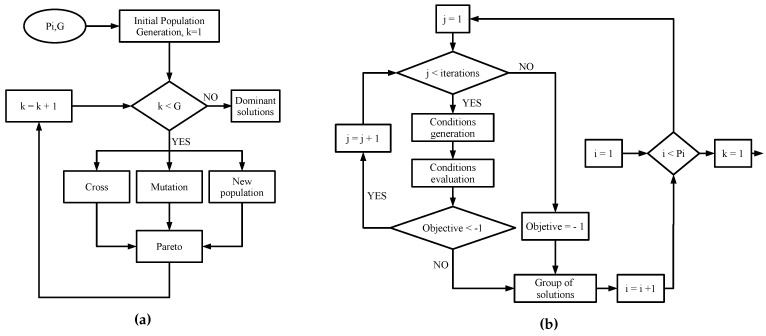
(**a**) Genetic algorithm for dominant solution generation. (**b**) Algorithm for initial population generation.

Non-linear optimization functions like fmincon are not suitable to optimize cycle operation because they fall in local optima rather than global optima. Therefore, an optimization algorithm based on a genetic meta-heuristic strategy (see Figure 3) was used to maximize simultaneous cooling and power production. Within this algorithm, a random initial population was generated (see Figure 3) by combining a set of boiler temperatures, rectifier temperatures, and pressure ratios from Table 4. Then, initial population was randomly crossed and mutated; meanwhile, a new population was generated. All generated solutions were filtered through the Pareto optimization criterion to obtain a set of dominant solutions.

## 3. Results and Discussion

**Figure 4:**
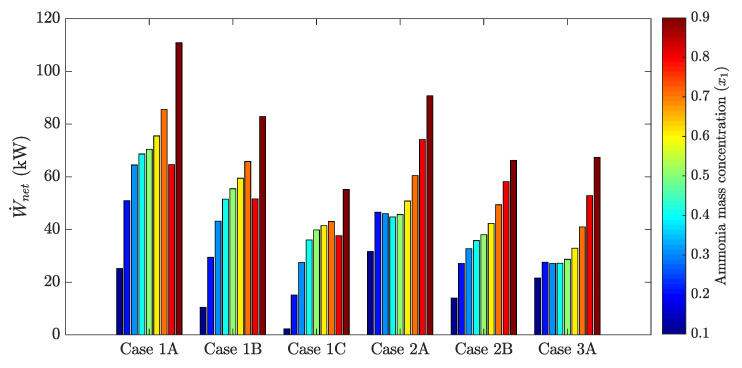
Optimum net power output for the simple pressure Goswami cycle.

**Figure 5:**
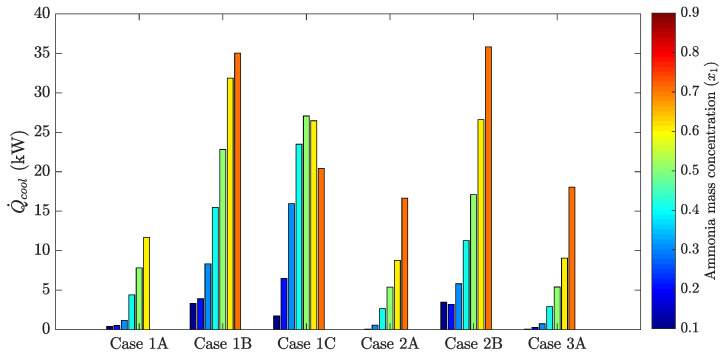
Optimum cooling output for the simple pressure Goswami cycle.

**Figure 6:**
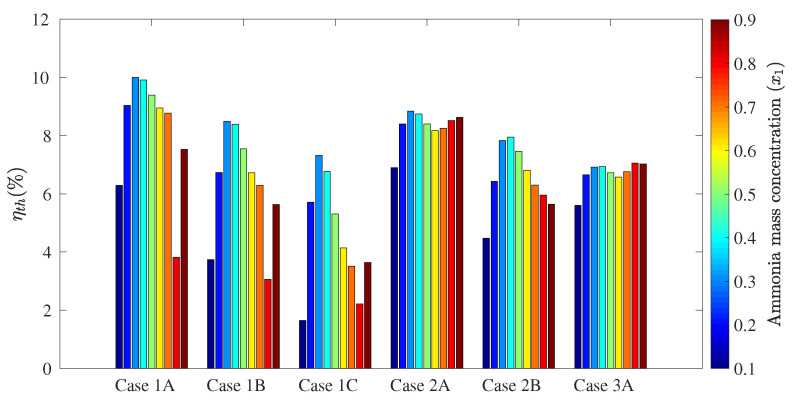
Optimum effective first law efficiency for the simple pressure Goswami cycle.

**Figure 7:**
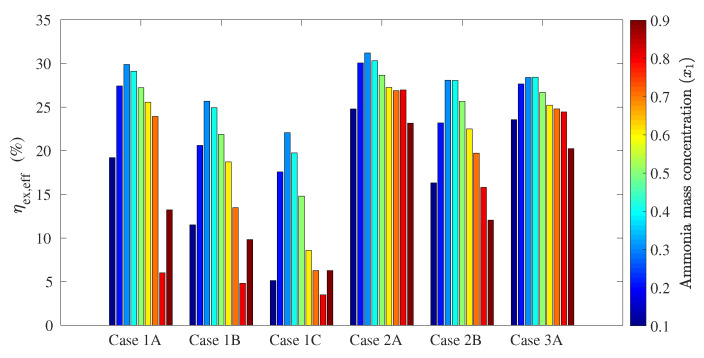
Optimum effective exergy efficiency for the simple pressure Goswami cycle.

**Figure 8:**
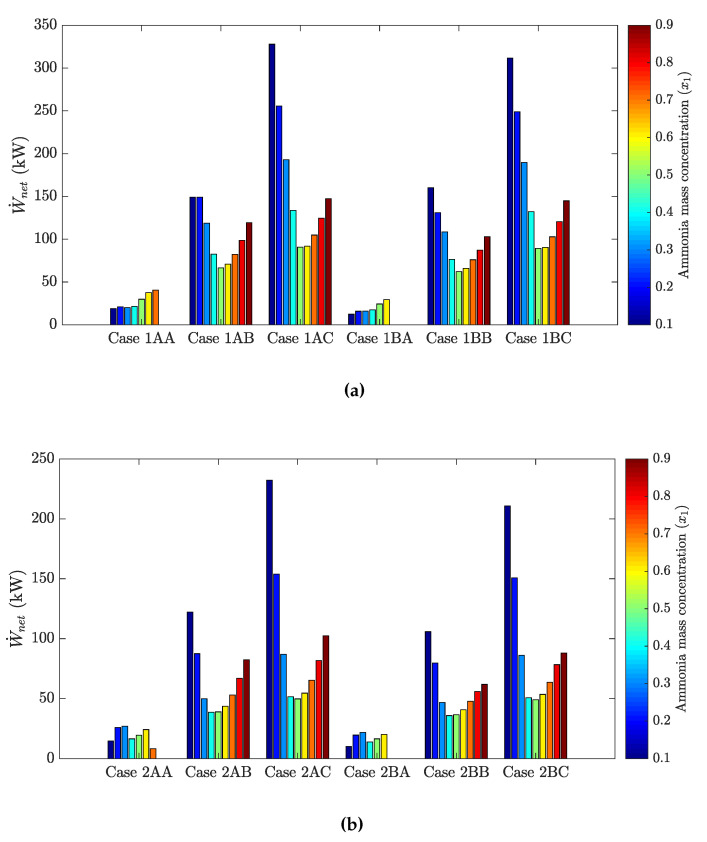
Optimum net power output for the dual pressure Goswami cycle. (**a**) Boiler temperature: 150 °C. (**b**) Boiler temperature: 120 °C.

**Figure 9:**
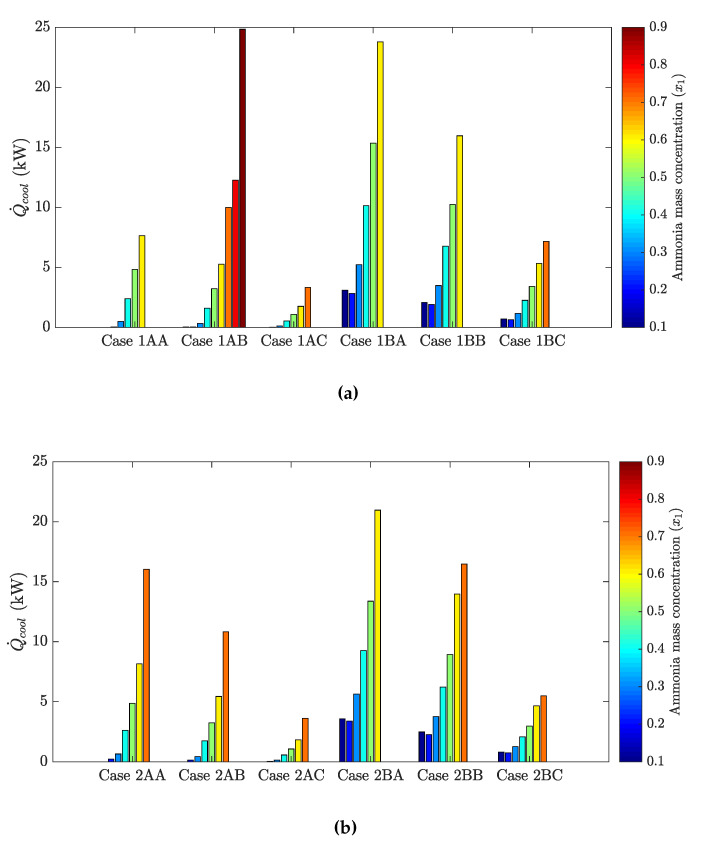
Optimum cooling output for the dual-pressure Goswami cycle. (**a**) Boiler temperature: 150 °C. (**b**) Boiler temperature: 120 °C.

**Figure 10:**
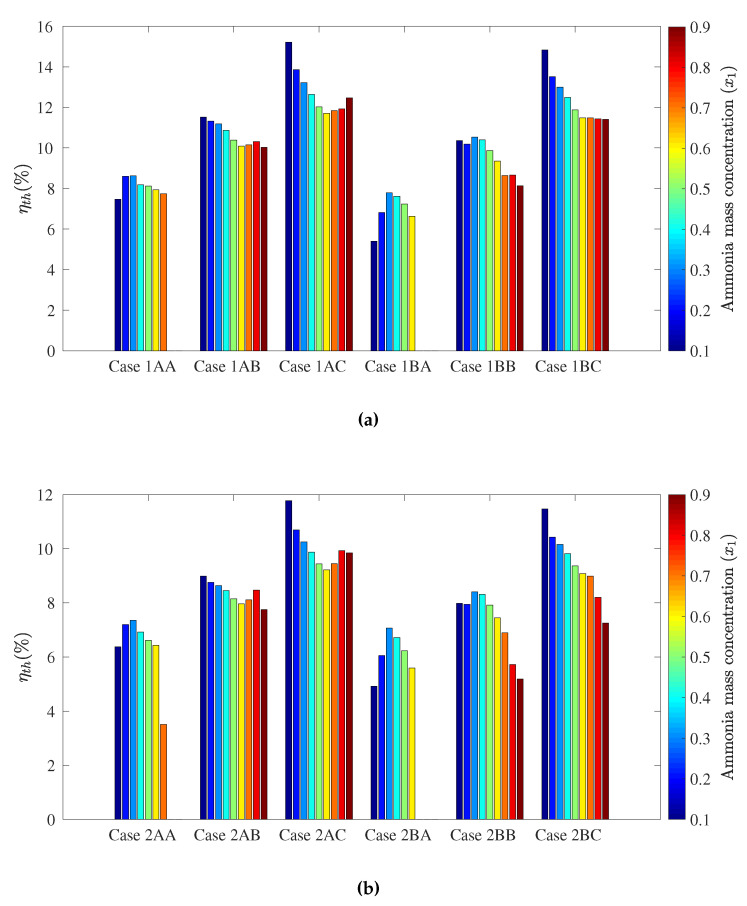
Optimum effective first law efficiency for the dual pressure Goswami cycle. (**a**) Boiler temperature: 150 °C. (**b**) Boiler temperature: 120 °C.

**Figure 11:**
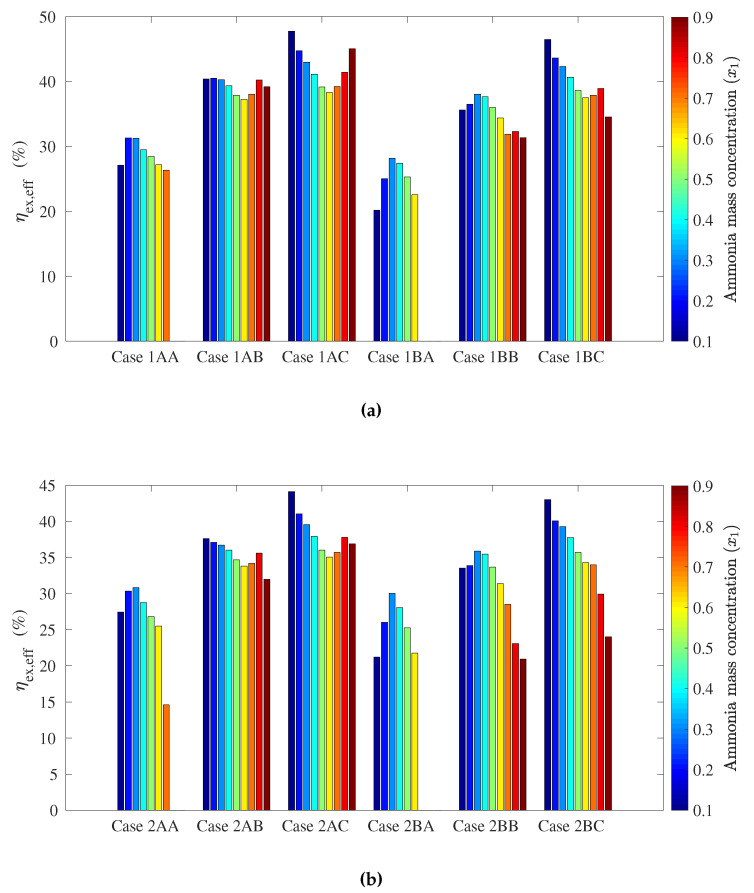
Optimum effective exergy efficiency for the dual-pressure Goswami cycle. (**a**) Boiler temperature: 150 °C. (**b**) Boiler temperature: 120 °C.

The following figures show the optimum performance metric values for each configuration. Each bar in Figure 4, Figure 5, Figure 6 and Figure 7 represents the maximum value for each combination in Table 1 when turbine inlet pressure in the single-pressure configuration was varied between the corresponding bubble and dew pressures. Similarly, Figure 8, Figure 9, Figure 10 and Figure 11 show the maximum performance metric for each combination in Table 3 when cycle pressures in the dual-pressure configuration were varied between the corresponding bubble and dew pressures. Each loop in the dual-pressure Goswami cycle had its own pressure range because each loop worked under different boiler temperatures. A summary of the optimum pressure ratios is found in Table A1, Table A2, Table A3, Table A4, Table A5 and Table A6 in Appendix A.1, and Table A7, Table A8, Table A9, Table A10, Table A11, Table A12, Table A13, Table A14, Table A15, Table A16, Table A17 and Table A18 in Appendix A.2.

### 3.1. Single-Pressure Goswami Cycle

Figure 4 depicts the maximum net power output obtained for each combination of boiler temperature, rectifier temperature, and absorber ammonia mass fraction in Table 1 when the cycle high pressure was varied between the boiler bubble and dew pressures. Results showed that rectifier temperature and absorber ammonia mass fraction has a significant effect on maximum net power output. The increase of these parameters boosted maximum net power output as it increased the optimum cycle high pressure, i.e., the pressure that achieved the maximum net power output, and increased vapor production in the rectifier column [18]. On the other hand, maximum net power output was improved when the boiler and rectifier temperatures were simultaneously increased (Cases 1A and 2A), but a high boiler temperature with a low rectifier temperature (Cases 1C and 2B) reduced the maximum net power output as it hindered the vapor production in the rectifier column.

Figure 5 shows the optimum cooling output as a function of rectifier and boiler temperatures and absorber ammonia mass fraction. For fixed boiler conditions, cooling capacity increased as rectifier temperature decreased. At higher boiler temperatures, ammonia mass fraction in Stream 8 leaving the rectifier dropped, and less cooling effect was achieved. Optimum cooling capacity was for a 0.7-kg NH_3_/kg solution, but above this limit, no cooling capacity can be achieved.

Figure 6 presents the optimum effective first law efficiency as a function of rectifier and boiler temperature and the ammonia mass fraction of the absorber. In the case of the effective first law efficiency, the effect of cooling power was remarkable. Simultaneous cooling and net power output was possible only in part of the operation pressure range, with a significant contribution to effective first law efficiency increase. Therefore, simultaneous power and cooling led to higher optimum effective first law efficiencies at absorber ammonia mass fractions from a 0.3–0.7-kg NH_3_/kg solution. Above 0.7 kg NH_3_/kg, optimum effective first law efficiencies were lower as no cooling output was obtained.

Effective exergy efficiency is presented in Figure 7. Results showed that maximum effective exergy efficiency was improved when the boiler and rectifier temperatures were decreased (Cases 2A, 2B, and 2C), and the absorber ammonia mass fraction ranged between a 0.2-and 0.5-kg NH_3_/kg solution. It can be inferred from Figure 4 and Figure 5 that the increase of the cycle cooling capacity reduced effective exergy efficiency as it increased entropy generation due to finite-difference heat transfer in the refrigeration unit. This trend can also be observed in Cases 1B and 1C, where the operation conditions that maximized cooling capacity also decreased effective exergy efficiency when the absorber mass fraction was below a 0.8-kg NH_3_/kg solution.

### 3.2. Dual-Pressure Goswami Cycle

Figure 8 shows optimum net power output for the proposed dual-pressure configuration of the Goswami cycle as a function of absorber ammonia mass fraction, boiler and rectifier temperature, and split ratio. It can be seen from Figure 8 that a higher power output was obtained when the split ratio was increased, leading to higher mass flow rates in the high-pressure loop. In the dual pressure cycle, the high-pressure loop was the high-temperature loop, leading to higher specific turbine work output. Therefore, net power output was boosted as the high-pressure mass flow rate increased.

Another important result from Figure 8 is the trend of the absorber ammonia mass fraction when the high-pressure loop mass flow rate was augmented. As the high-pressure loop mass flow rate went up, higher optimum net power outputs were found at a lower absorber ammonia mass fractions due to the enthalpy values of the ammonia-water mixtures increasing by decreasing the ammonia mass fraction. In addition, the system’s low pressure was at a minimum. Therefore, the highest net power outputs were found for a 0.1-kg NH_3_/kg solution. However, as the absorber ammonia mass fraction went up to 0.9 kg NH_3_/kg solution, optimum boiler pressure also increased and reduced the vaporization of water component, yielding higher vapor concentration and power output [22]. Finally, it can also be seen that net power output in Figure 8a was lower than that in Figure 8b due to lower boiler and rectifiers temperatures in the high-pressure loop, which hindered the power production potential.

Figure 9 depicts cooling production in the cycle as a function of the absorber ammonia mass fraction, boiler and rectifier temperature, and split ratio. The trend in Figure 9 is opposite that in Figure 8. Higher cooling outputs required higher mass flow rates and lower rectifier temperatures in the mid-pressure loop, but also lower absorber ammonia mass fractions. However, as was mentioned above, these conditions will penalize net power production. This clearly shows the trade-off between cooling output and net power production.

Regarding effective first law efficiency, Figure 10 gathers the cooling and net power trends. Results showed that the split ratio contribution was significant to the effective first law efficiency. As the split ratio increased, higher effective first law efficiencies were reached due to more net power output being obtained in the high-pressure loop. Results also showed that reducing boiler and rectifier temperatures led to lower effective first law efficiencies in the cycle. In general, reducing rectifier temperature increased cooling capacity, but reduced net power output, which sacrificed effective first law efficiency [22].

Regarding the absorber ammonia mass fraction, there was not a unified trend in Figure 10. In Cases 1BA and 2BA, an increase in the absorber ammonia mass fraction yielded effective first law efficiency due to relatively low net power output, and the effective first law efficiency trend was dominated by the cooling capacity. In Cases 1AB, 1BC, 2AC, and 2BC, higher power outputs were found for a 0.1-kg NH_3_/kg solution. Then, as the absorber ammonia mass fraction increased, simultaneous power and cooling occurred, and effective first law efficiency went up, but not as high as the ones for a 0.1-kg NH_3_/kg solution. Finally, Figure 10 shows that the highest efficiencies were found in Cases 1AC and 1BC, which had the highest net power output in Figure 8.

Effective exergy efficiency is presented in Figure 11. The maximum effective first law efficiency values were found for Cases 1AC, 1BC, 2AC, and 2BC. This means that the increase of ${\dot{m}}_{3}/{\dot{m}}_{2}$ improved the effective exergy efficiency in the dual-pressure configuration as a higher mass flow rate flowed throughout the high-pressure turbine, which increased the power output. In addition, Figure 11 also shows that higher boiler temperatures (Cases 1AC and 1BC) developed higher maximum effective exergy efficiencies, but at lower rectifier temperatures in the mid-pressure loop. Finally, lower absorber ammonia mass fractions, between a 0.1- and 0.2-kg NH_3_/kg solution, were required to maximize effective exergy efficiency.

### 3.3. Pareto Optimization Results

**Figure 12:**
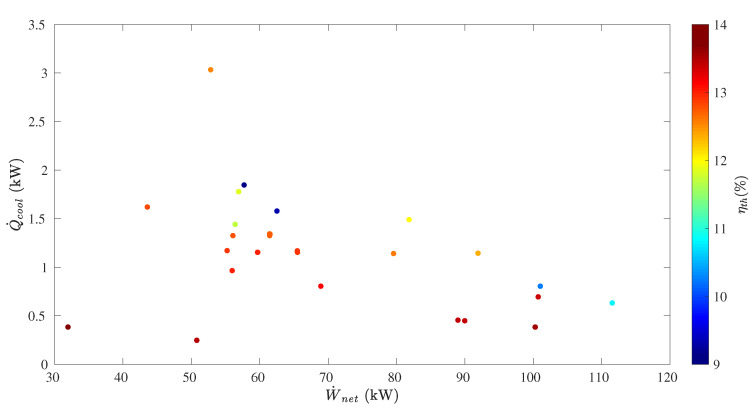
Genetic algorithm-based optimization results for the dual-pressure Goswami cycle.

Pareto results in Figure 12 show the optimum dominant solutions obtained from the optimization algorithm. However, the goal of this optimization was to maximize simultaneous power and cooling production. Therefore, solutions with power-only and cooling-only production were not taken into account.

From the optimization results, it can be seen that optimum effective first law efficiency ranged between 9.1% and 13.7%. It is important to point out that maximum effective first law efficiency (13.7%) was found at the lowest net power (32.0 kW) and cooling (0.38 kW) outputs. However, a very close value of 13.6% effective first law efficiency was achieved at 100.3 kW of net power output and 0.38 kW of cooling capacity. From Table A19 in Appendix B, to maximize simultaneously net power output and effective first law efficiency, the high-pressure boiler and rectifier temperatures had to be increased up to 149.8 °C and 148.2 °C, respectively, while the mid-pressure boiler and rectifier temperatures had to be set to 114.2 °C and 79.4 °C, respectively. In addition, absorber ammonia mass fractions between a 0.1- and 0.2-kg NH_3_/kg solution and ${\dot{m}}_{3}/{\dot{m}}_{2}$ between 0.45 and 0.62 were required.

The highest cooling capacity (3.0 kW) led to a 12.5% effective first law efficiency and 52.9 kW net power output. From Table A19 in Appendix B, the boiler and rectifier temperatures in the high-pressure loop and the boiler temperature in the mid-pressure loop were the same ones mentioned above. However, the rectifier temperature in the mid-pressure loop had to be decreased to 65.9 °C to achieve the highest cooling output. Therefore, to maximize simultaneously power and cooling production and achieve higher effective first law efficiency values, power production could be increased, but cooling capacity had to be reduced.

### 3.4. Comparison between the Cycles

Figure 8a shows that the dual-pressure configuration was able to produce up to 327.8 kW of net power output for Case 1AC, 311.6 kW for Case 1BC, 232.3 kW for Case 2AC, and 210.8 kW for Case 2BC. On the other hand, Figure 4 shows that the single-pressure configuration achieved up to 110.8 kW for Case 1A, 90.77 for Case 2A, and 82.87 kW for Case 1B. As in [18], none of these cases involved cooling production in the single- and dual-pressure configuration. Therefore, the dual-pressure Goswami cycle developed higher power output than the single-pressure cycle.

Demirkaya et al. [18] showed that no cooling capacity was obtained for boiler temperatures above 100 °C due to the turbine outlet temperature being above 40.5 °C and the rectifier not being active. From Figure 5, the single-pressure configuration developed up to 35.8 kW of cooling output for Case 2B (120 °C boiler temperature) and 35.03 kW for Case 1B (150 °C boiler temperature). From Figure 9, the dual-pressure configuration achieved up to 23.8 kW of cooling output for Case 1BA (150 °C temperature in the high-pressure boiler) and 21.0 kW for Case 2BA (120 °C temperature in the high-pressure boiler). However, effective first law efficiency was 5.4% in Case 2B, and 3.9% in Case 1B, while it was 5.4% for Case 2BA, and 5.9% for Case 1BA. Hence, the single-pressure configuration maximized cooling output, while the dual-pressure configuration achieved less cooling output, but both configurations showed very low values of effective first law efficiencies when cooling output was maximized.

Figure 11 shows that the dual-pressure Goswami cycle achieved up to 47.7% maximum effective exergy efficiency for Case 1AC, 46.5% for Case 1BC, 44.1% for Case 2AC, and 43.0% for Case 2BC. On the other hand, Figure 7 shows that the single-pressure configuration developed up to 29.9% effective exergy efficiency for Case 1A and 31.2% for Case 2A. Therefore, the dual-pressure configuration improved effective exergy efficiency when this efficiency was maximized.

### 3.5. Comparison with Other Cycles

Ogrisek et al. [23] presented a thermodynamic study of a Kalina cycle for waste heat recovery from a 124 °C heat source. Their study showed that the Kaline cycle can achieve a net conversion efficiency of 15.4% when a 20 °C cooling source is used. Thus, the dual-pressure configuration and the Kaline cycle have similar conversion efficiencies when they are working under these conditions, but the Goswami cycle produces an additional cooling output that cannot be found in the Kalina cycle. A study from Ayou et al. [24] introduced a single-stage combined absorption power and refrigeration cycle with series flow (SSAPRC-S) and a two-stage combined absorption power and refrigeration cycle with series flow (TSAPRC-S). They showed that the TSAPRC-S and the SSAPRC-S had a better performance than the single-pressure Goswami cycle by achieving an effective first law efficiency of 16.8% and 14.6%, respectively, at a 220 °C desorber temperature. However, the dual-pressure Goswami cycle achieved an effective first law efficiency of 15.2% for a boiler temperature of 150 °C. Therefore, the dual-pressure configuration was more efficient than the SSAPRC-S cycle, and its efficiency was very close to the efficiency in the TSAPRC-S cycle, but at lower boiler temperatures.

Astolfi et al. [25] carried out a thermodynamic study to maximize the performance of an organic Rankine cycle (ORC), when using heat source temperatures between 120 and 180 °C. Their study showed that a supercritical ORC that used organic fluids with a critical temperature slightly lower than the heat source temperature achieved first law efficiencies between 7.5% and 11.5%, which was below the maximum effective first law efficiency achieved by the dual-pressure configuration cycle (15.2% for power-only output and 13.7% for simultaneous power and cooling).

Table 5 shows a summary of some power and cooling applications that can be found in the literature. From this table, the performance of the dual-pressure configuration shows an interesting potential in terms of first law efficiency when it is compared to other ammonia-water combined power and cooling cycles. The single-pressure and the dual-pressure configuration achieved higher effective first law efficiency than the GAX-absorption refrigeration cycle when the boiler temperature was 155 °C. As mentioned above, the dual-pressure Goswami cycle developed a maximum effective first law efficiency of 15.2%, which was below the ones for the Rankine cycle with ejector refrigeration cycles in Table 5. However, as boiler temperatures in [26,27] were above 200 °C, the dual-pressure configuration can still achieve higher efficiencies if the boiler temperature is increased up to these temperature levels.

## 4. Conclusions

A theoretical analysis of a single-pressure and dual-pressure combined power and cooling cycle was conducted to find out the maximum performance of the cycle when it utilized thermal heat sources up to 150 °C. The effect of cycle parameters and cycle configurations on the performance of the system in terms of net power and cooling output, as well as effective first law and exergy efficiencies was determined. The following conclusions were obtained:

- The single-pressure configuration achieved a higher net power output by increasing the absorber ammonia mass fraction, as well as rectifier and boiler temperatures. However, higher boiler and rectifier temperatures decreased the cooling output.
- In the single-pressure configuration, simultaneous power and cooling led to higher optimum effective first law efficiencies at absorber ammonia mass fractions from 0.3–0.7 kg NH_3_/kg solution. Above 0.7 kg NH_3_/kg, effective first law efficiencies were lower as no cooling output was obtained.
- The addition of a high-pressure loop in the Goswami cycle increased net power output up to 327.8 kW, while the single-pressure configuration achieved up to 110.8 kW. However, for these values, the cooling effect was null.
- The single-pressure configuration maximized cooling output, up to 35.8 kW, while the dual-pressure configuration achieved less cooling output, up to 23.8 kW. Both configurations showed very low values of effective first law efficiencies, up to 5.9%, when cooling output was maximized.
- The energy conversion process in the dual-pressure configuration increased effective exergy efficiency. The dual-pressure configuration reached up to 47.7% of effective exergy efficiency in Case 1A, and eight of the twelve tested cases showed effective exergy efficiency values above 30%, while the single-pressure configuration achieved only up to 31.2%.
- Optimization results showed that optimum cycle effective first law efficiency ranged between 9.1% and 13.7%, showing the maximum effective first law efficiency at the lowest net power (32 kW) and cooling (0.38 kW) outputs. A very close value of 13.6% for effective first law efficiency was obtained when net power was 100 kW and and cooling capacity was 0.38 kW.

## Figures and Tables

**Table 1:** Operation conditions of the single-pressure Goswami cycle (see Figure 1).

Case	$T_{5}$ (°C)	$T_{4}$ (°C)	$x_{1}$ (kg NH_3_/kg sol)
1A	150	120	0.1–0.9
1B	150	100	0.1–0.9
1C	150	80	0.1–0.9
2A	120	100	0.1–0.9
2B	120	80	0.1–0.9
3A	100	80	0.1–0.9

**Table 2:** Cycle parameters assumed for the theoretical study [18].

Parameter	Value	Units
Pinch point	10	°C
Reference temperature	25	°C
Reference pressure	1	bar
Absorber temperature	35	°C
Second law efficiency of refrigeration $\eta_{\mathrm{II},\mathrm{ref}}$ [12]	30%	
Recovery heat exchanger effectiveness ε	85%	
Isentropic turbine efficiency $\eta_{t}$	85%	
Minimum turbine exit vapor quality	90%	
Isentropic pump efficiency $\eta_{pump}$	85%	

**Table 3:** Operation conditions of the dual-pressure Goswami cycle (see Figure 2).

Case	$T_{7}$ (°C)	$T_{13}$ (°C)	$T_{8}$ (°C)	$T_{14}$ (°C)	${\dot{m}}_{3}/{\dot{m}}_{2}$	$x_{1}$ (kg NH_3_/kg sol)
1AA	150	120	150	100	0.1	0.1–0.9
1AB	150	120	150	100	0.4	0.1–0.9
1AC	150	120	150	100	0.8	0.1–0.9
1BA	150	120	150	80	0.1	0.1–0.9
1BB	150	120	150	80	0.4	0.1–0.9
1BC	150	120	150	80	0.8	0.1–0.9
2AA	120	100	120	80	0.1	0.1–0.9
2AB	120	100	120	80	0.4	0.1–0.9
2AC	120	100	120	80	0.8	0.1–0.9
2BA	120	100	120	60	0.1	0.1–0.9
2BB	120	100	120	60	0.4	0.1–0.9
2BC	120	100	120	60	0.8	0.1–0.9

**Table 4:** Range of variable values for the proposed configuration.

	$x_{1}$	${\dot{m}}_{3}/{\dot{m}}_{2}$	${\mathrm{rp}}_{\mathrm{low}}$	${\mathrm{rp}}_{\mathrm{high}}$	$T_{7}$ (°C)	$T_{13}$ (°C)	$T_{8}$ (°C)	$T_{14}$ (°C)
**Lower limit**	0.1	0.1	1.2	1.5	80	60	80	50
**Upper limit**	0.9	0.8	2.0	4.0	150	120	150	80

**Table 5:** Summary of combined power and cooling cycles with an ammonia-water mixture as the working fluid from the literature. SSAPRC-S, single-stage combined absorption power and refrigeration cycle with series flow; TSAPRC-S, two-stage combined absorption power and refrigeration cycle with series flow.

Cycle Type	Ref.	Boiler (°C)	Condenser (°C)	$\eta_{\mathrm{carnot}}\;\left(\%\right)$	$\eta_{I}\left(\%\right)$	$\eta_{\mathrm{II}}\left(\%\right)$
GAX+ Absorption Ref.	[28] ${}^{*}$	155	28	30	11.9	-
Rankine + Ejector Ref.	[27] ${}^{*}$	212	25	39	20.9	35.8
	[26] ${}^{*}$	350	40.3	23.7	18.6	42.0
Kalina + Absorption Ref.	[29] ${}^{**}$	159	27	31	26	-
	[30] ${}^{*}$	160	25	32	11.1	-
	[31] ${}^{*}$	200	25	37	16.4	48.3
SSAPRC-S	[24] ${}^{*}$	220	30	48	14.6	-
TSAPRC-S	[24] ${}^{*}$	220	30	39	16.8	-

* Theoretical; ** Experimental.

## Abbreviations

The following abbreviations and symbols are used in this manuscript:

$x_{1}$	Absorber ammonia mass fraction (kg NH_3_/kg solution)
$\dot{E}$	Exergy rate (kW)
${\dot{E}}_{d}$	Exergy destruction (kW)
${\dot{E}}_{qj}$	Exergy input due to heat transfer (kW)
$e_{f}$	Stream specific exergy (kJ/kg)
$\dot{Q_{j}}$	Heat transfer input (kW)
$\dot{W}$	Power (kW)
*T*	Temperature (°C or K)
*V*	Velocity (m/s)
*m*	Mass flow rate (kg/s)
*h*	Specific enthalpy (kJ/kg)
*s*	Specific entropy (kJ/kg·K)
*g*	Gravity (m/s${}^{2}$)
*z*	Vertical position with respect to the ground (m)
$rp_{\mathrm{low}}$	Pressure ratio across the mid-pressure turbine ($P_{2}/P_{1}$)
$rp_{\mathrm{high}}$	Pressure ratio across the high-pressure turbine ($P_{5}/P_{3}$)

**Subscripts**	
cf	Chilled fluid
cv	Control volume
cool	Cooling
II	Second law
i	Inlet
e	Outlet
ex	Exergy
eff	Effective value
*h*	Heat
hs	Heat source
net	Net
*o*	Reference
th	First law
ref	Refrigeration

**Greek symbols**	
η	Efficiency

## Appendix A. Optimum Pressure Ratio

### Appendix A.1. Single-Pressure Goswami Cycle

The main result data regarding the parametric study of the single-pressure Goswami cycle can be found from Table A1, Table A2, Table A3, Table A4, Table A5, Table A6.

**Table A1:** Parametric study results for the single-pressure Goswami cycle: Case 1A.

	rp			
$x_{1}$	**Optimum** ${\dot{W}}_{\mathrm{net}}$	**Optimum** ${\dot{Q}}_{\mathrm{cool}}$	**Optimum** $\eta_{\mathrm{th}}$	**Optimum** $\eta_{\mathrm{ex},\mathrm{eff}}$
0.1	25.84	36.48	32.37	32.93
0.2	11.31	24.8	19.44	20.18
0.3	6.28	17.9	13.35	14.19
0.4	4.8	14.08	9.72	10.28
0.5	4.21	10.71	7.4	7.84
0.6	3.98	9.17	6.06	6.34
0.7	3.83	7.22	5.43	6.47
0.8	1.85	1.46	1.85	1.85
0.9	3.19	1.51	3.19	3.19

**Table A2:** Parametric study results for the single-pressure Goswami cycle: Case 1B.

	rp			
$x_{1}$	**Optimum** ${\dot{W}}_{\mathrm{net}}$	**Optimum** ${\dot{Q}}_{\mathrm{cool}}$	**Optimum** $\eta_{\mathrm{th}}$	**Optimum** $\eta_{\mathrm{ex},\mathrm{eff}}$
0.1	28.83	29.76	33.12	33.49
0.2	13.16	17.78	20.18	20.92
0.3	7.79	15.54	14.53	15.03
0.4	5.5	11.69	10.56	10.99
0.5	4.21	9.16	7.62	7.95
0.6	3.7	7.47	5.58	6.24
0.7	3.45	6.18	4.49	5.24
0.8	1.85	1.01	1.85	1.85
0.9	2.93	1.01	3.06	3.09

**Table A3:** Parametric study results for the single-pressure Goswami cycle: Case 1C.

	rp			
$x_{1}$	**Optimum** ${\dot{W}}_{\mathrm{net}}$	**Optimum** ${\dot{Q}}_{\mathrm{cool}}$	**Optimum** $\eta_{\mathrm{th}}$	**Optimum** $\eta_{\mathrm{ex},\mathrm{eff}}$
0.1	32	32.37	34.05	34.24
0.2	15.38	18.52	20.92	21.11
0.3	9.14	12.51	14.36	14.87
0.4	5.92	9.3	9.58	9.72
0.5	4.1	6.52	5.97	6.3
0.6	3.22	4.83	4.07	4.36
0.7	2.79	3.92	3.07	3.17
0.8	1.85	1.01	1.85	1.85
0.9	2.2	1.01	2.23	2.23

**Table A4:** Parametric study results for the single-pressure Goswami cycle: Case 2A.

	rp			
$x_{1}$	**Optimum** ${\dot{W}}_{\mathrm{net}}$	**Optimum** ${\dot{Q}}_{\mathrm{cool}}$	**Optimum** $\eta_{\mathrm{th}}$	**Optimum** $\eta_{\mathrm{ex},\mathrm{eff}}$
0.1	10.11	18.7	15.14	15.66
0.2	4.64	13.49	9.89	10.4
0.3	3.25	10.23	7.52	8
0.4	3.26	8.25	6.01	6.44
0.5	3.1	6.61	4.96	5.39
0.6	2.99	5.83	4.38	4.63
0.7	2.95	5.95	4.14	4.39
0.8	3.11	5.22	4.13	4.39
0.9	3.27	4.6	3.91	4.54

**Table A5:** Parametric study results for the single-pressure Goswami cycle: Case 2B.

	rp			
$x_{1}$	**Optimum** ${\dot{W}}_{\mathrm{net}}$	**Optimum** ${\dot{Q}}_{\mathrm{cool}}$	**Optimum** $\eta_{\mathrm{th}}$	**Optimum** $\eta_{\mathrm{ex},\mathrm{eff}}$
0.1	12.1	14.09	15.66	16.08
0.2	5.77	9.47	10.19	10.61
0.3	4.03	8.58	8.2	8.49
0.4	3.43	6.79	6.53	6.87
0.5	3.1	5.68	5.25	5.54
0.6	2.93	5.01	4.32	4.51
0.7	2.83	4.2	3.58	3.7
0.8	2.73	3.43	2.92	3.37
0.9	2.52	2.86	2.81	2.81

**Table A6:** Parametric study results for the single-pressure Goswami cycle: Case 3A.

	rp			
$x_{1}$	**Optimum** ${\dot{W}}_{\mathrm{net}}$	**Optimum** ${\dot{Q}}_{\mathrm{cool}}$	**Optimum** $\eta_{\mathrm{th}}$	**Optimum** $\eta_{\mathrm{ex},\mathrm{eff}}$
0.1	5.33	10.76	8.62	9.02
0.2	3.06	7.82	6.04	6.37
0.3	2.7	5.96	4.96	5.27
0.4	2.61	5.19	4.39	4.62
0.5	2.46	4.37	3.67	3.92
0.6	2.42	4	3.32	3.5
0.7	2.46	4	3.16	3.38
0.8	2.54	3.43	3.25	3.34
0.9	2.55	2.88	2.88	2.88

### Appendix A.2. Dual-Pressure Goswami Cycle

The main result data regarding the parametric study of the single-pressure Goswami cycle can be found from Table A7, Table A8, Table A9, Table A10, Table A11, Table A12, Table A13, Table A14, Table A15, Table A16, Table A17, Table A18.

**Table A7:** Parametric study results for the dual-pressure Goswami cycle: Case 1AA.

	Optimum ${\dot{W}}_{\mathrm{net}}$		Optimum ${\dot{Q}}_{\mathrm{cool}}$		Optimum $\eta_{\mathrm{th}}$		Optimum $\eta_{\mathrm{ex},\mathrm{eff}}$	
$x_{1}$	${\mathrm{rp}}_{\mathrm{low}}$	${\mathrm{rp}}_{\mathrm{high}}$	${\mathrm{rp}}_{\mathrm{low}}$	${\mathrm{rp}}_{\mathrm{high}}$	${\mathrm{rp}}_{\mathrm{low}}$	${\mathrm{rp}}_{\mathrm{high}}$	${\mathrm{rp}}_{\mathrm{low}}$	${\mathrm{rp}}_{\mathrm{high}}$
0.1	14.41	2.41	-	-	15.45	2.24	15.45	2.24
0.2	9.78	2.39	13.49	1.96	9.78	2.39	10.71	2.17
0.3	8.2	2.36	10.23	2.1	8.2	2.36	8.2	2.36
0.4	6.79	2.32	8.25	2.02	6.79	2.32	6.79	2.32
0.5	5.18	2.29	6.54	1.95	5.18	2.29	5.18	2.29
0.6	4.38	2.25	5.64	1.91	4.38	2.25	4.38	2.25
0.7	4.45	2.24	-	-	4.45	2.24	4.45	2.24

**Table A8:** Parametric study results for the dual-pressure Goswami cycle: Case 1AB.

	Optimum ${\dot{W}}_{\mathrm{net}}$		Optimum ${\dot{Q}}_{\mathrm{cool}}$		Optimum $\eta_{\mathrm{th}}$		Optimum $\eta_{\mathrm{ex},\mathrm{eff}}$	
$x_{1}$	${\mathrm{rp}}_{\mathrm{low}}$	${\mathrm{rp}}_{\mathrm{high}}$	${\mathrm{rp}}_{\mathrm{low}}$	${\mathrm{rp}}_{\mathrm{high}}$	${\mathrm{rp}}_{\mathrm{low}}$	${\mathrm{rp}}_{\mathrm{high}}$	${\mathrm{rp}}_{\mathrm{low}}$	${\mathrm{rp}}_{\mathrm{high}}$
0.1	10.11	2.07	-	-	13.46	2.07	14.3	2.07
0.2	3.81	2.39	13.49	1.96	9.27	1.96	9.58	1.96
0.3	2.67	1.85	10.23	2.1	7.23	1.85	7.42	1.85
0.4	1.8	1.72	8.25	2.02	5.93	1.72	6.01	1.72
0.5	2.6	1.62	6.61	1.62	4.96	1.62	4.96	1.62
0.6	2.74	1.57	5.83	1.57	4.32	1.57	4.38	1.57
0.7	2.83	1.58	5.95	1.58	4.08	1.58	4.08	1.58
0.8	3.37	1.13	5.79	1.13	4.19	1.13	4.19	1.13
0.9	3.1	1.28	5.01	1.28	3.1	2.57	3.1	2.57

**Table A9:** Parametric study results for the dual-pressure Goswami cycle: Case 1AC.

	Optimum ${\dot{W}}_{\mathrm{net}}$		Optimum ${\dot{Q}}_{\mathrm{cool}}$		Optimum $\eta_{\mathrm{th}}$		Optimum $\eta_{\mathrm{ex},\mathrm{eff}}$	
$x_{1}$	${\mathrm{rp}}_{\mathrm{low}}$	${\mathrm{rp}}_{\mathrm{high}}$	${\mathrm{rp}}_{\mathrm{low}}$	${\mathrm{rp}}_{\mathrm{high}}$	${\mathrm{rp}}_{\mathrm{low}}$	${\mathrm{rp}}_{\mathrm{high}}$	${\mathrm{rp}}_{\mathrm{low}}$	${\mathrm{rp}}_{\mathrm{high}}$
0.1	8.65	2.41	-	-	12.94	2.07	14.41	2.07
0.2	3.81	2.39	13.49	1.96	8.86	1.96	9.47	1.96
0.3	2.67	1.85	10.23	1.85	7.03	1.85	7.42	1.85
0.4	1.28	2.32	8.25	1.72	5.93	1.72	6.1	1.72
0.5	1.81	1.62	6.61	1.62	4.96	1.62	5.18	1.62
0.6	2.55	1.57	5.83	1.57	4.32	1.57	4.44	1.57
0.7	2.64	1.58	5.95	1.58	4.08	1.58	4.14	1.58
0.8	3.68	1.13	-	-	2.92	2.27	3.05	2.27
0.9	2.05	2.57	-	-	2.81	2.57	2.98	2.57

**Table A10:** Parametric study results for the dual-pressure Goswami cycle: Case 1BA.

	Optimum ${\dot{W}}_{\mathrm{net}}$		Optimum ${\dot{Q}}_{\mathrm{cool}}$		Optimum $\eta_{\mathrm{th}}$		Optimum $\eta_{\mathrm{ex},\mathrm{eff}}$	
$x_{1}$	${\mathrm{rp}}_{\mathrm{low}}$	${\mathrm{rp}}_{\mathrm{high}}$	${\mathrm{rp}}_{\mathrm{low}}$	${\mathrm{rp}}_{\mathrm{high}}$	${\mathrm{rp}}_{\mathrm{low}}$	${\mathrm{rp}}_{\mathrm{high}}$	${\mathrm{rp}}_{\mathrm{low}}$	${\mathrm{rp}}_{\mathrm{high}}$
0.1	14.41	2.41	14.41	2.41	16.71	2.07	16.71	2.07
0.2	9.78	2.39	9.78	2.39	10.71	2.17	10.71	2.17
0.3	8.2	2.36	8.58	2.36	8.2	2.36	8.2	2.36
0.4	6.79	2.32	6.79	2.32	6.79	2.32	6.79	2.32
0.5	5.18	2.29	5.61	2.29	5.18	2.29	5.18	2.29
0.6	4.38	2.25	5.14	1.91	4.38	2.25	4.38	2.25

**Table A11:** Parametric study results for the dual-pressure Goswami cycle: Case 1BB.

	Optimum ${\dot{W}}_{\mathrm{net}}$		Optimum ${\dot{Q}}_{\mathrm{cool}}$		Optimum $\eta_{\mathrm{th}}$		Optimum $\eta_{\mathrm{ex},\mathrm{eff}}$	
$x_{1}$	${\mathrm{rp}}_{\mathrm{low}}$	${\mathrm{rp}}_{\mathrm{high}}$	${\mathrm{rp}}_{\mathrm{low}}$	${\mathrm{rp}}_{\mathrm{high}}$	${\mathrm{rp}}_{\mathrm{low}}$	${\mathrm{rp}}_{\mathrm{high}}$	${\mathrm{rp}}_{\mathrm{low}}$	${\mathrm{rp}}_{\mathrm{high}}$
0.1	10.11	2.07	14.09	2.41	10.11	2.07	14.3	2.07
0.2	4.22	2.17	9.47	2.39	9.37	1.96	9.68	1.96
0.3	2.67	1.85	8.58	2.36	7.62	1.85	7.71	1.85
0.4	1.8	1.72	6.79	2.32	6.18	1.72	6.27	1.72
0.5	2.6	1.62	5.68	1.95	5.11	1.62	5.18	1.62
0.6	2.68	1.57	5.01	1.91	4.32	1.57	4.32	1.57
0.7	2.7	1.58	-	-	3.01	1.91	3.01	1.91
0.8	2.92	1.13	-	-	2.92	2.27	2.92	2.27
0.9	2.11	2.57	-	-	2.11	2.57	2.11	2.57

**Table A12:** Parametric study results for the dual-pressure Goswami cycle: Case 1BC.

	Optimum ${\dot{W}}_{\mathrm{net}}$		Optimum ${\dot{Q}}_{\mathrm{cool}}$		Optimum $\eta_{\mathrm{th}}$		Optimum $\eta_{\mathrm{ex},\mathrm{eff}}$	
$x_{1}$	${\mathrm{rp}}_{\mathrm{low}}$	${\mathrm{rp}}_{\mathrm{high}}$	${\mathrm{rp}}_{\mathrm{low}}$	${\mathrm{rp}}_{\mathrm{high}}$	${\mathrm{rp}}_{\mathrm{low}}$	${\mathrm{rp}}_{\mathrm{high}}$	${\mathrm{rp}}_{\mathrm{low}}$	${\mathrm{rp}}_{\mathrm{high}}$
0.1	10.11	2.07	14.09	2.41	12.84	2.07	14.41	2.07
0.2	3.81	2.39	9.47	2.39	8.86	1.96	9.47	1.96
0.3	2.67	1.85	8.58	2.36	7.13	1.85	7.42	1.85
0.4	1.28	2.32	6.79	2.32	5.93	1.72	6.18	1.72
0.5	1.74	1.62	5.68	1.95	5.03	1.62	5.11	1.62
0.6	2.49	1.57	5.01	1.91	4.32	1.57	4.44	1.57
0.7	2.64	1.58	4.2	2.24	3.95	1.58	3.51	1.91
0.8	2.03	2.27	-	-	2.54	2.27	2.54	2.27
0.9	2	2.57	-	-	2.05	2.57	2.05	2.57

**Table A13:** Parametric study results for the dual-pressure Goswami cycle: Case 2AA.

	Optimum ${\dot{W}}_{\mathrm{net}}$		Optimum ${\dot{Q}}_{\mathrm{cool}}$		Optimum $\eta_{\mathrm{th}}$		Optimum $\eta_{\mathrm{ex},\mathrm{eff}}$	
$x_{1}$	${\mathrm{rp}}_{\mathrm{low}}$	${\mathrm{rp}}_{\mathrm{high}}$	${\mathrm{rp}}_{\mathrm{low}}$	${\mathrm{rp}}_{\mathrm{high}}$	${\mathrm{rp}}_{\mathrm{low}}$	${\mathrm{rp}}_{\mathrm{high}}$	${\mathrm{rp}}_{\mathrm{low}}$	${\mathrm{rp}}_{\mathrm{high}}$
0.1	7.95	1.97	-	-	9.02	1.72	9.02	1.72
0.2	4.32	1.97	7.82	1.65	5.97	1.65	6.3	1.65
0.3	3.45	1.95	5.96	1.95	4.89	1.61	5.14	1.61
0.4	4.39	1.94	5.19	1.74	4.39	1.94	4.39	1.94
0.5	3.72	1.91	4.37	1.69	3.72	1.91	3.72	1.91
0.6	3.32	1.89	4	1.66	3.32	1.89	3.69	1.66
0.7	4.09	1.65	4.09	1.65	4.09	1.65	4.09	1.65

**Table A14:** Parametric study results for the dual-pressure Goswami cycle: Case 2AB.

	Optimum ${\dot{W}}_{\mathrm{net}}$		Optimum ${\dot{Q}}_{\mathrm{cool}}$		Optimum $\eta_{\mathrm{th}}$		Optimum $\eta_{\mathrm{ex},\mathrm{eff}}$	
$x_{1}$	${\mathrm{rp}}_{\mathrm{low}}$	${\mathrm{rp}}_{\mathrm{high}}$	${\mathrm{rp}}_{\mathrm{low}}$	${\mathrm{rp}}_{\mathrm{high}}$	${\mathrm{rp}}_{\mathrm{low}}$	${\mathrm{rp}}_{\mathrm{high}}$	${\mathrm{rp}}_{\mathrm{low}}$	${\mathrm{rp}}_{\mathrm{high}}$
0.1	4.4	1.97	-	-	8.01	1.72	8.42	1.72
0.2	2.34	1.65	7.82	1.65	5.77	1.65	5.97	1.65
0.3	1.32	1.61	5.96	1.95	4.77	1.61	4.96	1.61
0.4	2.21	1.54	5.19	1.74	4.16	1.54	4.28	1.54
0.5	2.26	1.47	4.37	1.69	3.57	1.47	3.67	1.47
0.6	2.28	1.43	4	1.66	3.28	1.43	3.28	1.43
0.7	2.28	1.45	4	1.65	2.99	1.45	2.99	1.45
0.8	2.41	1.47	-	-	2.94	1.47	2.94	1.47
0.9	2.14	1.68	-	-	2.14	1.88	2.14	1.88

**Table A15:** Parametric study results for the dual-pressure Goswami cycle: Case 2AC.

	Optimum ${\dot{W}}_{\mathrm{net}}$		Optimum ${\dot{Q}}_{\mathrm{cool}}$		Optimum $\eta_{\mathrm{th}}$		Optimum $\eta_{\mathrm{ex},\mathrm{eff}}$	
$x_{1}$	${\mathrm{rp}}_{\mathrm{low}}$	${\mathrm{rp}}_{\mathrm{high}}$	${\mathrm{rp}}_{\mathrm{low}}$	${\mathrm{rp}}_{\mathrm{high}}$	${\mathrm{rp}}_{\mathrm{low}}$	${\mathrm{rp}}_{\mathrm{high}}$	${\mathrm{rp}}_{\mathrm{low}}$	${\mathrm{rp}}_{\mathrm{high}}$
0.1	4.4	1.97	-	-	7.75	1.72	8.48	1.72
0.2	1.94	1.97	7.82	1.65	5.57	1.65	5.97	1.65
0.3	1.32	1.61	5.96	1.78	4.64	1.61	4.89	1.61
0.4	1.87	1.54	5.19	1.74	3.99	1.54	4.22	1.54
0.5	2.11	1.47	4.37	1.69	3.52	1.47	3.67	1.47
0.6	2.15	1.43	4	1.43	3.28	1.43	3.28	1.43
0.7	2.19	1.45	4	1.45	2.99	1.45	2.81	1.65
0.8	2.27	1.47	-	-	2.94	1.47	2.94	1.65
0.9	2.14	1.68	-	-	2.14	1.88	2.14	1.88

**Table A16:** Parametric study results for the dual-pressure Goswami cycle: Case 2BA.

	Optimum ${\dot{W}}_{\mathrm{net}}$		Optimum ${\dot{Q}}_{\mathrm{cool}}$		Optimum $\eta_{\mathrm{th}}$		Optimum $\eta_{\mathrm{ex},\mathrm{eff}}$	
$x_{1}$	${\mathrm{rp}}_{\mathrm{low}}$	${\mathrm{rp}}_{\mathrm{high}}$	${\mathrm{rp}}_{\mathrm{low}}$	${\mathrm{rp}}_{\mathrm{high}}$	${\mathrm{rp}}_{\mathrm{low}}$	${\mathrm{rp}}_{\mathrm{high}}$	${\mathrm{rp}}_{\mathrm{low}}$	${\mathrm{rp}}_{\mathrm{high}}$
0.1	7.95	1.97	7.95	1.97	9.02	1.72	9.02	1.72
0.2	4.32	1.97	4.98	1.97	6.43	1.65	6.63	1.65
0.3	3.45	1.95	5.08	1.95	5.21	1.61	5.4	1.61
0.4	4.39	1.94	4.39	1.94	4.39	1.94	4.85	1.74
0.5	3.72	1.91	3.77	1.91	3.72	1.91	3.72	1.91
0.6	3.23	1.89	3.32	1.89	3.23	1.89	3.23	1.89

**Table A17:** Parametric study results for the dual-pressure Goswami cycle: Case 2BB.

	Optimum ${\dot{W}}_{\mathrm{net}}$		Optimum ${\dot{Q}}_{\mathrm{cool}}$		Optimum $\eta_{\mathrm{th}}$		Optimum $\eta_{\mathrm{ex},\mathrm{eff}}$	
$x_{1}$	${\mathrm{rp}}_{\mathrm{low}}$	${\mathrm{rp}}_{\mathrm{high}}$	${\mathrm{rp}}_{\mathrm{low}}$	${\mathrm{rp}}_{\mathrm{high}}$	${\mathrm{rp}}_{\mathrm{low}}$	${\mathrm{rp}}_{\mathrm{high}}$	${\mathrm{rp}}_{\mathrm{low}}$	${\mathrm{rp}}_{\mathrm{high}}$
0.1	4.73	1.85	7.41	1.97	8.15	1.72	8.48	1.72
0.2	2.34	1.65	4.98	1.97	5.97	1.65	6.17	1.65
0.3	1.32	1.61	5.08	1.95	4.96	1.61	5.08	1.61
0.4	2.21	1.54	4.22	1.94	4.22	1.54	4.33	1.54
0.5	2.26	1.47	3.77	1.91	3.62	1.47	3.72	1.47
0.6	2.28	1.43	3.32	1.89	3.14	1.43	3.19	1.43
0.7	1.97	1.65	2.63	1.85	2.63	1.45	2.63	1.45
0.8	1.69	1.83	-	-	1.69	1.83	1.69	1.83
0.9	1.48	1.88	-	-	1.48	1.88	1.48	1.88

**Table A18:** Parametric study results for the dual-pressure Goswami cycle: Case 2BC.

	Optimum ${\dot{W}}_{\mathrm{net}}$		Optimum ${\dot{Q}}_{\mathrm{cool}}$		Optimum $\eta_{\mathrm{th}}$		Optimum $\eta_{\mathrm{ex},\mathrm{eff}}$	
$x_{1}$	${\mathrm{rp}}_{\mathrm{low}}$	${\mathrm{rp}}_{\mathrm{high}}$	${\mathrm{rp}}_{\mathrm{low}}$	${\mathrm{rp}}_{\mathrm{high}}$	${\mathrm{rp}}_{\mathrm{low}}$	${\mathrm{rp}}_{\mathrm{high}}$	${\mathrm{rp}}_{\mathrm{low}}$	${\mathrm{rp}}_{\mathrm{high}}$
0.1	4.73	1.85	7.41	1.97	7.75	1.72	8.48	1.72
0.2	1.94	1.97	4.98	1.97	5.57	1.65	5.97	1.65
0.3	1.07	1.95	5.08	1.95	4.71	1.61	4.96	1.61
0.4	1.87	1.54	4.22	1.94	4.05	1.54	4.22	1.54
0.5	2.11	1.47	3.77	1.91	3.52	1.47	3.67	1.47
0.6	2.24	1.43	3.32	1.89	3.14	1.43	3.23	1.43
0.7	1.92	1.65	2.63	1.85	2.59	1.65	2.63	1.65
0.8	1.74	1.83	-	-	1.74	1.83	1.74	1.83
0.9	1.4	1.88	-	-	1.4	1.88	1.4	1.88

## Appendix B. Pareto Optimization Data

The main result data regarding the optimization study of the dual-pressure Goswami cycle can be found in Table A19.

**Table A19:** Optimization results for the dual-pressure Goswami cycle.

$T_{7}$ (°C)	$T_{13}$ (°C)	$T_{8}$ (°C)	$T_{14}$ (°C)	${\mathrm{rp}}_{\mathrm{low}}$	${\mathrm{rp}}_{\mathrm{high}}$	$x_{1}$	${\dot{m}}_{3}/{\dot{m}}_{2}$	${\dot{W}}_{\mathrm{net}}$	$\eta_{\mathrm{th}}$	${\dot{Q}}_{\mathrm{cool}}$
149.84	114.24	140.78	65.93	7.13	1.75	0.2	0.55	57.76	9.1	1.84
149.84	114.24	140.78	65.93	7.13	1.75	0.2	0.62	62.57	9.33	1.57
149.86	117.03	148.18	70	5.83	1.93	0.2	0.62	101.07	10.22	0.8
149.84	117.03	148.18	70	5.83	1.93	0.2	0.7	111.59	10.79	0.63
133.56	110.67	138.86	75.48	10.89	1.5	0.1	0.45	56.46	11.7	1.44
149.84	116.3	148.18	75.33	8.65	1.57	0.2	0.48	56.97	11.82	1.77
149.86	116.3	148.18	73.16	8.21	1.5	0.2	0.61	81.89	11.99	1.49
149.84	116.51	148.18	73.16	8.21	1.5	0.2	0.7	91.96	12.33	1.14
149.86	114.24	148.18	65.93	7.18	1.58	0.28	0.62	52.88	12.54	3.03
149.84	116.3	148.18	75.33	8.65	1.5	0.2	0.67	79.62	12.56	1.14
149.84	116.51	148.18	66.17	9.46	1.5	0.2	0.62	61.54	12.7	1.34
149.86	116.3	148.18	66.17	9.46	1.5	0.2	0.62	61.5	12.71	1.32
149.86	116.3	148.18	65.93	9.46	1.5	0.2	0.62	61.49	12.71	1.34
149.86	116.3	148.18	66.17	9.46	1.57	0.2	0.62	56.13	12.75	1.32
149.84	116.51	148.18	65.94	9.46	1.65	0.22	0.62	43.61	12.82	1.62
149.86	114.24	148.18	65.93	9.46	1.57	0.2	0.62	55.28	12.91	1.17
149.86	116.3	148.18	66.17	9.46	1.5	0.2	0.67	65.56	12.92	1.15
149.86	116.3	148.18	65.93	9.46	1.5	0.2	0.67	65.55	12.92	1.17
149.86	116.3	148.18	66.17	9.46	1.57	0.2	0.67	59.75	12.98	1.15
149.84	116.3	148.18	66.17	9.46	1.57	0.2	0.67	59.73	12.98	1.15
149.86	114.24	148.18	75.33	9.46	1.57	0.2	0.62	56.02	13.01	0.96
149.86	116.3	148.18	79.45	9.46	1.5	0.2	0.7	68.98	13.14	0.8
149.84	116.3	148.18	66.17	15.43	1.5	0.1	0.62	100.76	13.38	0.69
149.86	114.24	148.18	79.45	15.43	1.5	0.1	0.55	89.03	13.4	0.45
149.86	114.24	148.18	79.45	15.43	1.5	0.1	0.55	90.02	13.42	0.45
133.56	101.06	138.86	75.48	10.89	1.5	0.1	0.45	50.84	13.47	0.24
149.86	114.24	148.18	79.45	15.43	1.5	0.1	0.62	100.34	13.54	0.38
149.84	114.24	148.18	79.45	15.43	1.5	0.1	0.62	100.31	13.56	0.38
149.84	114.24	148.18	79.45	15.43	1.97	0.1	0.62	32.01	13.71	0.38
